# From self-awareness to social savvy: how intrapersonal skills shape interpersonal competence in university students

**DOI:** 10.3389/fpsyg.2024.1469746

**Published:** 2024-09-02

**Authors:** Ida Merlin J., Prabakar Soubramanian

**Affiliations:** Department of Social Sciences, School of Social Sciences and Languages, Vellore Institute of Technology, Vellore, India

**Keywords:** emotional intelligence, intrapersonal competence, interpersonal competence, university students, psychological well-being

## Abstract

**Introduction:**

The extant study was conducted over a cross-sectional period and aimed to assess the effect of intrapersonal on the interpersonal dimensions of Emotional Intelligence among University Students.

**Methods:**

A literature survey was carried out, and the study’s hypotheses were framed. Utilising a standardised Emotional Intelligence Scale, a widely accepted and validated measurement tool in the field, for measurement, the survey was disseminated in digital and physical formats. The researchers employed the snowball sampling technique to distribute the questionnaires and recruit volunteers for the study. The data collection period spanned from August 2023 through September 2023. The demographic information of the individuals was described using the SPSS 25 software, while the dataset for the personal and social competencies was analysed using the SmartPLS software.

**Results and discussion:**

The research reveals a statistically significant association between the variables under investigation. Specifically, there exists a negative correlation between Motivation and Social Skills, as well as between Self-regulation and Social Awareness. These findings open up exciting opportunities for future research, inspiring further exploration into the development of intrapersonal and interpersonal competencies among students.

## Introduction

A critical aspect that influences a human’s ability to make decisions, control emotions and manage behaviour is emotional intelligence. It is a concept that gained popularity for its significance in practical applications and academic research in various fields after Daniel Goleman published his book on Emotional Intelligence in 1995. It is “the ability to recognise, understand, manage and utilise emotions effectively in oneself and others” (Mayer et al., 1999). The competencies of Emotional intelligence are widely divided into intrapersonal and interpersonal competence. Though they are widely distinct, these competencies are interconnected and help improve the emotional and social functioning of society. There are two perspectives of emotional intelligence: ability emotional intelligence (Mayer et al., 2008) and trait emotional intelligence (Petrides et al., 2007). Several models of emotional intelligence, such as those proposed by Goleman (1995), Mayer and Salovey (1997), and Bar-On (2006), were developed to understand the concept of emotional intelligence with sub-competencies. Previous studies, including those by Petrides and Furnham (2000) and Schutte et al. (2001), have shown that the development of intrapersonal skills and interpersonal skills impacts various outcome variables. This raises the question: Can the intrapersonal skills of emotional intelligence, as discussed in Goleman (2001), have an influence on the interpersonal skills of emotional intelligence? To answer the above question, the article’s key objective is to find if the different intrapersonal skills of emotional intelligence impact the interpersonal skills of emotional intelligence, as these skills are some of the components that contribute to the psychological well-being of individuals. The article provides a comprehensive overview of the two broad components of EI and emphasises the implications of EI, which is essential for the psychological well-being of Students.

## Intrapersonal competence of emotional intelligence

Intrapersonal Competence makes an individual introspect and manage one’s emotions. It is the heart of emotional intelligence. The three components that are the core of intrapersonal competence are self-awareness, self-regulation and self-motivation. These are important not only for emotional well-being but also for the personal development of individuals (Ștefan, 2022). It helps deal with the everyday challenges of life (Bar-On, 2006). It has lower-order emotional intelligence abilities, which are emotional perception, emotional understanding and emotional expression. Emotional perception is a skill that espies and interprets the body language, the volume and pitch of voice, and facial expressions in oneself and in others (Krumhuber et al., 2023). Emotional expression is the ability to convey emotional information through verbal and non-verbal cues, such as recognising emotional triggers, understanding emotional dynamics, the awareness of emotional complexity and cognitive empathy aligning with different contexts (Seidel et al., 2010). Comprehending the causes and the consequences of the emotions expressed is emotional understanding. These basic abilities are required for the emotional functioning of an individual. Apart from the fact that the above abilities stipulate the individual to reflect and grow by managing their emotions, communicating effectively with empathetic interactions, and building relationships, it also leads to better professional development by contributing to better leadership (Saha et al., 2023; Lee et al., 2023) and conflict resolution skills (Mercader-Rubio et al., 2023). Accurate emotional reasoning and emotional information processing are integral for intrapersonal skills. Self-awareness has three sub-competencies, emotional self-awareness which allows individuals to understand their feelings and have a direct impact on their decisions and actions. Accurate self-assessment helps one understand their strengths and weaknesses. An individual can evaluate oneself and recognise areas of improvement on their own, which would help one develop personally as well. A strong sense of self-worth helps stem self-confidence, which is the realistic perception of one’s ability to express oneself and make decisions. Daniel Goleman, in his book on Emotional Intelligence, identifies that self, awareness is the foundation for developing the other components of EI, which include social awareness and social skills (Goleman, 1995) and that self-reflection, which is a key aspect of self-awareness is linked to social cognition, which involves understanding the emotions of others during social interactions (Silvia and Duval, 2001). Mayer and Salovey (1997) highlighted that self-awareness develops social competence, which includes both social awareness and social skills. This theory aligns with the idea that self-awareness, or the understanding of one’s own mental states, is essential for understanding others’ perspectives (Woodruff and Premack, 1978), which is a fundamental aspect of social awareness and social skills. Hence, the following hypothesis was framed to test in the current study.

*H1*: Self-awareness influences the social awareness of students.

*H2*: Self-awareness influences the social skills of students.

Self-regulation is defined as a person’s ability to manage one’s internal impulses. It maintains integrity by disrupting negative emotions and helps adapt to various environments and situations. It is a combination of the subskills of self-control, conscientiousness, adaptability, trustworthiness, and innovation. Self-control is the key component of self-regulation, which keeps the impulsive nature in check, helps manage stress and behaves acceptably socially (Wenzel et al., 2024). Trustworthiness and integrity help maintain standards of ethical behaviour and be consistent in one’s values and principles (Christison and Murray, 2023). Taking responsibility by being reliable, diligent and organised and setting high standards for oneself is conscientiousness. The ability to adjust to new environments and be flexible and open to new ideas is adaptability (Boyar et al., 2023). When an individual is comfortable with new approaches and takes risks in exploring creative ideas and solutions in novel situations is innovative (Winton and Sabol, 2024). A research highlighted that individuals who regulate their emotions can understand and respond to the emotions of others better (Gross, 2002). Another study showed that emotional regulation had an impact on the quality of social interaction (Lopes et al., 2005). Eisenberg and Spinrad (2004) also emphasised how self-regulation impacted empathy, which is the key element of social awareness. They also further found that self-regulated individuals were better at listening and responding to information (Eisenberg et al., 2010), which is essential for effective social interactions. Students with better self-regulation also exhibited better social skills, which included cooperation and responsibility (McClelland et al., 2007). Hence, the following hypothesis was framed to test in the current study.

*H3*: Self-regulation influences the social awareness of students.

*H4*: Self-regulation influences the social skills of students.

Self-motivation helps one to be energetic and optimistic. These include achievement drive, the desire of an individual to attain a standard of excellence in life by setting challenging goals and being determined to achieve them. Commitment is a skill that helps one align with the group or organisational goals and be dedicated to achieving the objectives (Rahiman et al., 2020). When an individual is ready to take proactive steps and is a self-starter to prompt actions on opportunities, it shows that the individual takes initiative, which is a component of self-motivation (Kugbey et al., 2018; Venkatesh and Balaji, 2013). Motivation also includes being optimistic, a positive outlook on a person’s ability to overcome setbacks and obstacles and be resilient (Christie et al., 2007).

Although there is evidence that intrapersonal emotional intelligence improves both emotional and physical health (Resurreccion et al., 2014), some research indicates that having high EI, especially emotional awareness, may negatively affect the individual’s ability to handle emotionally significant situations (Martins et al., 2010). Self-Determination Theory emphasises that motivation is linked to better social understanding and higher levels of empathy (Ryan and Deci, 2000). They also argued that this empathy developed into strong social skills. This was further supported by another study that suggested that intrinsic motivation promoted prosocial behaviour (Gagné and Deci, 2005). Another study discussed how motivated individuals are more socially aware (Sansone and Thoman, 2005), and it enhanced empathy and social skills (Grant and Berry, 2011). Hence, based on the above literature, the following hypotheses were formulated to be tested.

*H5*: Motivation influences social awareness.

*H6*: Motivation influences social skills.

## Interpersonal competence of emotional intelligence

In the emotional intelligence context, interpersonal competence is the ability to manage social interactions by understanding and regulations one’s own emotions and others. This competence is momentous in education (Lindsey and Rice, 2015; Willmot and Colman, 2016), showing evidence for effective classroom management (Valente et al., 2019) and academic achievement (Mohzan et al., 2013; Costa and Faria, 2015; Parker et al., 2004). In psychology, emotional intelligence is linked to better communicative adequacy (Raeissi et al., 2023) and relationship-building (Schutte et al., 2001). In healthcare, there are studies showing that emotional intelligence directly influences better performance in their roles (Govindaraju, 2021).

The interpersonal competence of emotional intelligence has social awareness and social skills, which have multiple sub-competencies. Social awareness is being able to understand and empathise with the feelings and perspectives of others. In a social context, it is being aware of the cultural differences and the effect of one’s behaviour on other individuals and social groups by having empathy which requires a person to take an active interest in others and read the emotional cues, interests and desires of others and be compassionate and supportive toward them (Imperato and Strano-Paul, 2021; Austin et al., 2007). Organisational awareness is a social awareness that helps one understand social networking and power dynamics within an institution. It helps with effective collaboration and influences acceptable behaviour in formal and informal structures (Aamir, 2023). Offering service and support to others with the proactive ability to anticipate and meet the needs of others to build a positive culture within an institution is service orientation (Shafait and Huang, 2024).

*H7*: Social awareness influences the social skills of students.

Managing social interactions in an effective manner is a social skill. Influencing others by persuading and guiding them to achieve common goals is an integral part of social skills (Singh and Ryhal, 2023). It also includes being able to communicate actively, understand others’ emotional tones and express ideas clearly to others (Raeissi et al., 2023; Behera and Pani, 2014). Resolving disagreements and managing conflicts to find beneficial solutions to problems (Schlaerth et al., 2013) and being authentic in leading groups and motivating them to thrive is a skill of leadership (Miao et al., 2018). Social skills also require catalysing change for adaptability and building bonds (Goleman, 2001) for long-term maintenance of relationships to induce collaboration (Cox, 2011) and cooperation (Fernández-Berrocal et al., 2014) among individuals, which also helps enhance the team capabilities (Shafique and Naz, 2023) by creating group synergy and creates a more cohesive group.

**Figure 1:**
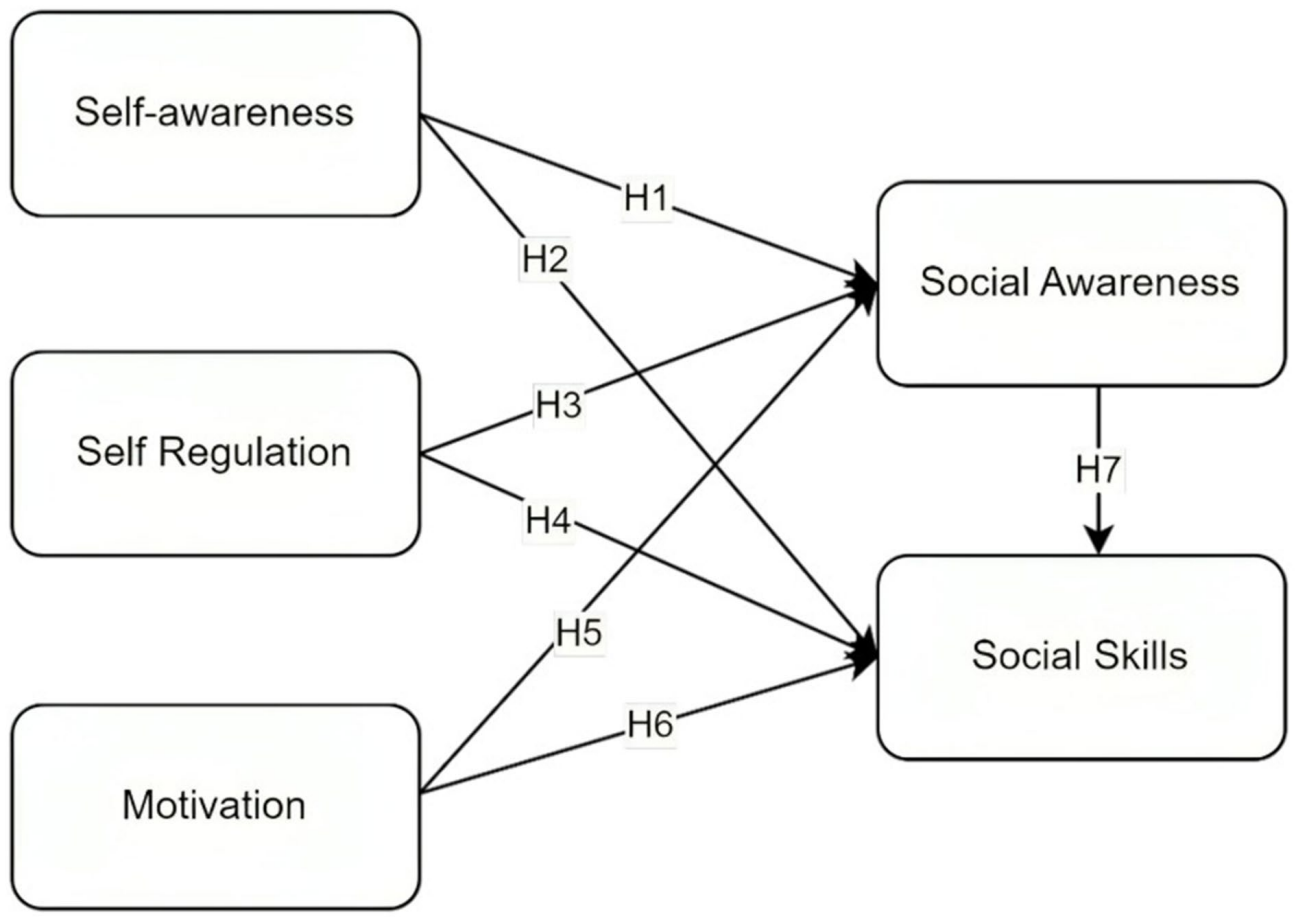
Proposed model.

The gap from the previous literature shows that though emotional intelligence as a whole has an impact on other variables, there is a lack of studies that show the impact of self-competence on the social competence of emotional intelligence, which holistically builds the psychological well-being of students. Figure 1 shows the proposed model aligning with the hypotheses formulated for the current study.

## Methodology

The study adopted the cross-sectional research design; hence, the data was collected from the participants at one point. A comprehensive literature research was undertaken, and a questionnaire tailored to the Indian participants was adapted from Dr. Shailendra Singh’s Emotional Intelligence Scale for the present study (Singh, 2004). A total of 352 students participated in the study using snowball sampling from the province of Vellore. Next, the hypotheses for the investigation were formulated. The data that was gathered was subsequently subjected to analysis utilising the SPSS and SMARTPLS tools. The subjects’ demographic information was analysed using SPSS 25 for descriptive statistics. Partial Least Squares Structural Equation Modelling (PLS-SEM) was employed to analyse and investigate the collected data. The main objective of this study was to assess the psychometric properties of the measuring instrument and to statistically examine the assumptions of the study model (Hair et al., 2019a,b). Given the innovative nature of this work in developing new structural pathways, the use of Partial Least Squares Structural Equation Modelling (PLS-SEM) was deemed appropriate. The measurement model was first verified, and afterwards, the structural model was validated using Smart-PLS version 4.0. The demographic information of the participants in the current study is shown elaborately in Table 1. The demographic information included were gender, age, religion, community, stream of education and family type to ensure that students from different socio-demographic backgrounds were included in the study and to get comprehensive data that is not monotonous in nature.

**Table 1:** Demographic classification of the participants.

Demographic variables		Frequency	%
Gender	Male	157	49.1
	Female	163	50.9
Age	20	100	31.3
	21	133	41.6
	22	87	27.2
Religion	Hindu	216	67.5
	Christian	77	24.1
	Muslim	27	8.4
Community	General	147	45.9
	OBC	136	42.5
	SC/ST	37	11.6
Stream of education	Arts and Science	132	41.3
	Engineering	108	33.8
	Management	23	7.2
	Medical	57	17.8
Family type	Extended family	24	7.5
	Joint family	104	32.5
	Nuclear family	176	55
	Single parent family	16	5

## Results

The PLS technique requires a measurement model evaluation to identify inaccurate indicator variables before assessing the structural model, according to Hair et al. (2019a,b). The reflecting measurement models’ composite reliability, Cronbach’s alpha, individual indicator reliability, commonality, and average variance extracted (AVE) are used to assess internal consistency, convergent validity, and commonality. The Fornell-Larcker criteria and HTMT tests assessed discriminant validity (Hair et al., 2019a,b).

**Figure 2:**
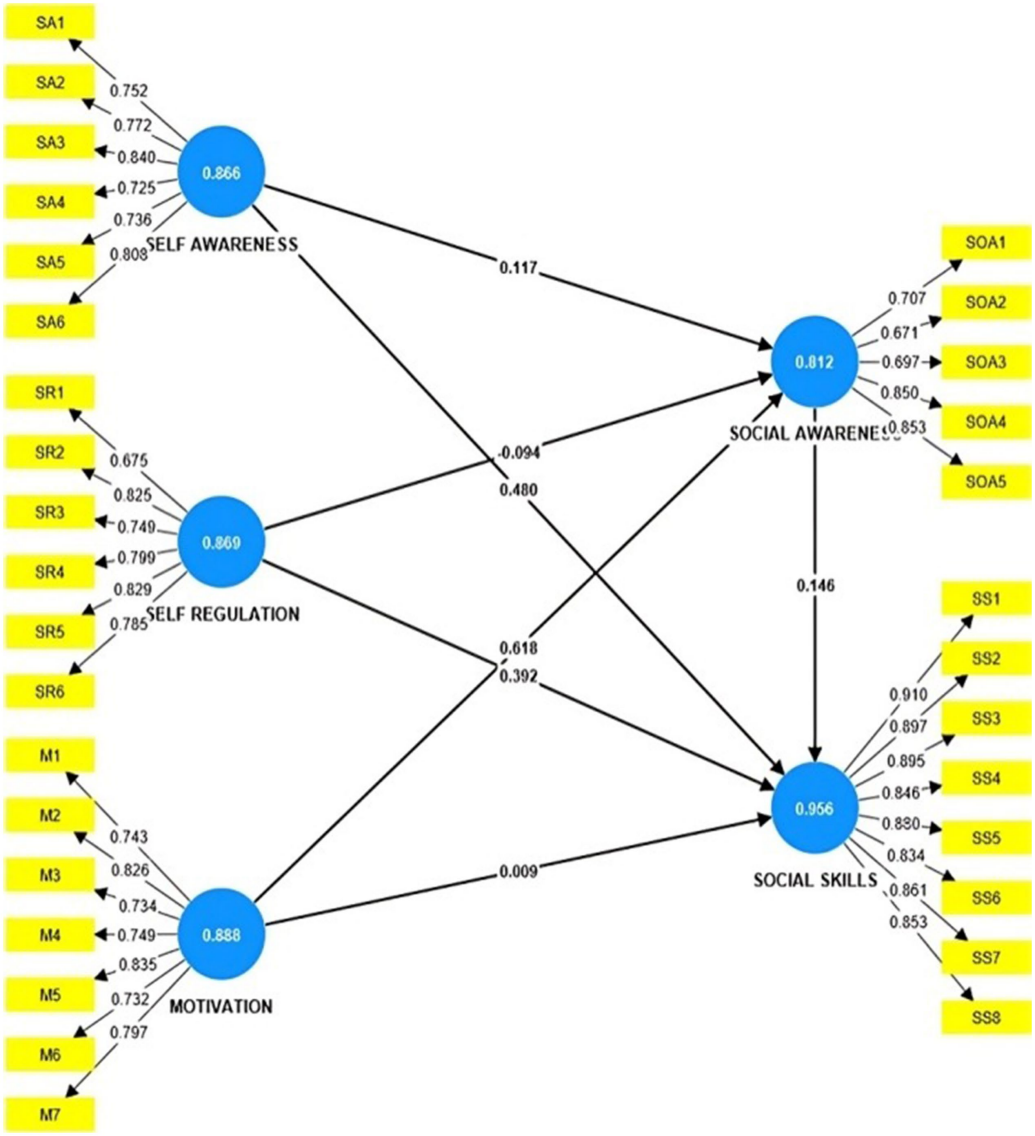
Measurement model using SMARTPLS.

Figure 2 shows the measurement model obtained using smart PLS. The inner model consists of the path coefficients and *p* values. The constructs consist of Cronbach’s alpha values. The outer model consists of the outer weights/loadings and *p* values.

In the individual item level, the loadings of the indicators and their commonality surpassed the threshold values of 0.7 and 0.5, respectively. The outer loading of SOA6 did not significantly contribute to its respective construct; therefore, it was removed. Furthermore, all constructs demonstrated AVE values above 0.50, confirming convergent validity. The composite reliability values for all the constructs were above the threshold of 0.70, indicating adequate internal consistency (Table 2).

**Table 2:** Convergent validity of the measurement model.

	Cronbach’s alpha	Composite reliability (rho_a)	Composite reliability (rho_c)	Average variance extracted (AVE)
Self-awareness	0.866	0.88	0.899	0.598
Self-regulation	0.869	0.876	0.902	0.606
Motivation	0.888	0.89	0.913	0.6
Social awareness	0.812	0.812	0.871	0.577
Social skills	0.956	0.963	0.962	0.761

Discriminant validity was assessed using the Fornell-Larcker criteria and the Heterotrait-Monotrait ratios of correlations. The Fornell-Larcker analysis reveals that the square root of the average variance extracted (AVE) for each construct surpasses the correlations with other constructs, as presented in Table 3. All constructs exhibited discriminant validity.

**Table 3:** Fornell-Larker criterion.

	Motivation	Self-awareness	Self-regulation	Social awareness	Social skills
Motivation	0.775				
Self-awareness	0.114	0.773			
Self-regulation	0.128	0.705	0.779		
Social awareness	0.619	0.121	0.068	0.76	
Social skills	0.204	0.775	0.741	0.236	0.872

The HTMT values for all variables were found to be below the threshold of 0.85, providing evidence in support of discriminant validity (refer to Table 4) (Voorhees et al., 2016).

**Table 4:** Discriminant validity—HTMT.

	Motivation	Self-awareness	Self-regulation	Social awareness	Social skills
Motivation					
Self-awareness	0.124				
Self-regulation	0.144	0.794			
Social awareness	0.722	0.154	0.124		
Social skills	0.213	0.828	0.782	0.265	

Discriminant validity is evaluated and reported when the loading on a certain construct surpasses the loadings on other constructs while considering the cross-loading values. The values of the constructs for all indicators were found to be greater than the values of their corresponding cross-loadings (see Table 5). After ensuring the fulfilment of convergent and discriminant validity, the structural model was evaluated.

**Table 5:** Cross loadings.

	Motivation	Self awareness	Self regulation	Social awareness	Social skills
M1	**0.743**	0.091	0.069	0.477	0.111
M2	**0.826**	0.059	0.088	0.442	0.138
M3	**0.734**	0.11	0.087	0.495	0.161
M4	**0.749**	0.091	0.129	0.418	0.167
M5	**0.835**	0.087	0.114	0.49	0.189
M6	**0.732**	0.113	0.093	0.476	0.16
M7	**0.797**	0.067	0.116	0.538	0.175
SA1	0.112	**0.752**	0.498	0.14	0.596
SA2	0.057	**0.772**	0.592	0.022	0.537
SA3	0.108	**0.84**	0.592	0.105	0.64
SA4	0.04	**0.725**	0.43	0.103	0.508
SA5	0.056	**0.736**	0.411	0.089	0.468
SA6	0.129	**0.808**	0.682	0.097	0.766
SOA1	0.522	0.039	−0.033	**0.707**	0.102
SOA2	0.392	0.18	0.137	**0.671**	0.242
SOA3	0.531	0.039	0.001	**0.697**	0.119
SOA4	0.423	0.11	0.069	**0.85**	0.217
SOA5	0.461	0.098	0.09	**0.853**	0.219
SR1	0.108	0.565	**0.675**	0.087	0.542
SR2	0.022	0.553	**0.825**	−0.031	0.586
SR3	0.071	0.531	**0.749**	0.038	0.481
SR4	0.122	0.486	**0.799**	0.037	0.511
SR5	0.111	0.542	**0.829**	0.052	0.622
SR6	0.154	0.6	**0.785**	0.12	0.676
SS1	0.214	0.707	0.769	0.241	**0.91**
SS2	0.207	0.718	0.757	0.231	**0.897**
SS3	0.236	0.74	0.767	0.252	**0.895**
SS4	0.137	0.653	0.504	0.202	**0.846**
SS5	0.2	0.693	0.767	0.195	**0.88**
SS6	0.114	0.607	0.485	0.158	**0.834**
SS7	0.135	0.63	0.505	0.171	**0.861**
SS8	0.138	0.631	0.49	0.172	**0.853**

The cross loadings of items against their factors have been marked in bold.

The output of the structural model, obtained by the application of bootstrapping with 5,000 samples, is presented in Table 6. The table displays seven hypotheses (H1, H2, H3, H4, H5, H6, H7) along with their corresponding T statistics and *p* values. The hypotheses H1, H2, H4, H5, and H7 have been accepted based on their *p*-values being less than 0.05. This suggests that there is a statistically significant relationship between self-awareness and social awareness (*β* value = 0.117, *p* value = 0.026), self-awareness and social skills (*β* value = 0.480, *p* value = 0), self-regulation and social skills (*β* value = 0.392, *p* value = 0), motivation and social awareness (*β* value = 0.618, *p* value = 0), and social awareness and social skills (*β* value = 0.146, *p* value = 0.002). However, the H3 and H6 hypotheses are deemed invalid as their respective *p* values (0.084 and 0.869) are above the significance level of 0.05. This suggests that there is insufficient statistical evidence to support the existence of a relationship between the variables.

**Table 6:** Path coefficients of the variables.

Hypotheses		Original sample (O)	Sample mean (M)	Standard deviation (STDEV)	T statistics (|O/STDEV|)	*p* values
H1	SELF AWARENESS - > SOCIAL AWARENESS	0.117	0.117	0.053	2.224	0.026
H2	SELF-AWARENESS - > SOCIAL SKILLS	0.481	0.481	0.056	8.523	0
H3	SELF REGULATION - > SOCIAL AWARENESS	−0.097	−0.097	0.056	1.73	**0.084**
H4	SELF REGULATION - > SOCIAL SKILLS	0.39	0.391	0.051	7.713	0
H5	MOTIVATION - > SOCIAL AWARENESS	0.62	0.626	0.041	14.993	0
H6	MOTIVATION - > SOCIAL SKILLS	0.008	0.006	0.048	0.165	**0.869**
H7	SOCIAL AWARENESS - > SOCIAL SKILLS	0.146	0.145	0.046	3.169	0.002

The hypothesis that have been rejected are marked in bold.

## Discussion

From the analysed data, it can be observed that self-awareness impacts social awareness. Though a similar study showed a negative correlation between the two (Merlin and Prabakar, 2024), the current study supports the theory and previous study results (Bratton et al., 2011). Self-awareness had a significant impact on social skills, showing the importance of self-awareness in the development of social skills among individuals. Self-awareness plays a pivotal role in one’s behaviour in the social setting. Self-awareness acts as a bridge to understanding others and how the social environment works and helps individuals to enhance their social self to receive better social support and build bonds. Self-regulation and Motivation negatively impacted the social skills of the individuals in the current study. This could be because self-regulation and motivation are skills that are focused on the self and do not necessarily contribute to the social self of a person. Previous studies support the result, showing that self-regulation did not impact the social skills of individuals (Merlin and Prabakar, 2024). Further in-depth research needs to be done to fathom why these self-competences do not impact social competencies of emotional intelligence. Different perspectives on why the intrapersonal competencies did not contribute to interpersonal competence development must also be considered. Self-regulation and social awareness influenced the social skills of the students. Previous studies also support the current findings on the importance of self-regulation (Pelco and Reed-Victor, 2007) and social awareness (Eisenberg et al., 2010; Urrila and Mäkelä, 2024) for social skills. The results also highlight the importance of the development of self-awareness, self-regulation and motivation. It is possible that the students are low in these competencies. Hence, these competencies do not have an impact on the interpersonal competencies.

EI is increasingly recognised as an important factor in influencing the psychological well-being of students. It boosts students’ academic performance (Bilimale et al., 2024; Swetha et al., 2023), interpersonal relationships (Bechter et al., 2023; Parker et al., 2021) and overall mental health (Xu and Choi, 2023; Augusto-Landa et al., 2024). Emotional Intelligence also serves as a crucial tool for managing stress (Ullah et al., 2023; Cajachagua Castro et al., 2023), have resilience (Sk and Halder, 2024; Nguyen et al., 2023; Hwang and Kim, 2023) which helps relieve from psychological distress (Abualruz et al., 2024). Some of the practical implications to enhance both intrapersonal and interpersonal emotional intelligence are introducing emotional intelligence integrated curriculum design in the institutions instead of treating it as an extracurricular subject. Personalised emotional learning plans that can help support the development of EI skills and mindful technology use programs, which is a novel approach to leveraging EI for psychological well-being. Peer support networks can also be created to empower each other’s emotional and psychological well-being.

The study had certain limitations. The data collected and analysed are only based on the responses from the self-report questionnaire. Given the fact that the concept of the study is exploratory within the emotional intelligence domain, further investigations can be done with support from qualitative data or observational methods to comprehensively understand the relationship between the intrapersonal and interpersonal competencies of emotional intelligence.

## Conclusion

The results from the present study show that certain intrapersonal competencies do not contribute to the development of interpersonal competence of emotional intelligence among students. Despite empirical evidence from various studies showing that interpersonal skills play a significant role in the development of interpersonal skills, the current study shows the negative impact of self-regulation on social awareness and motivation on social skills. This shows the need for further investigations on the dimensions of emotional intelligence used in the study. This could also be due to the overemphasis on the emotional competencies, that the other competencies are overlooked and not given importance. However, the results of the current study cannot be universally applicable because of the novelty of the findings. Further investigations should be done to check if similar results are obtained from the duplication of the concepts in the study.

Emotional intelligence has far-reaching implications for the psychological well-being of students. Learning to develop emotional intelligence not only enhances mental health but also provides the tools required for success in many spheres of life. Therefore, encouraging the psychological well-being of students and enabling them to realise their best potential depends on developing EI in learning environments.

## Data Availability

The raw data supporting the conclusions of this article will be made available by the authors, without undue reservation.
